# Paracrine-mediated rejuvenation of aged mesenchymal stem cells is associated with downregulation of the autophagy-lysosomal pathway

**DOI:** 10.1038/s41514-022-00091-0

**Published:** 2022-07-18

**Authors:** George Hung, Tamara Ashvetiya, Aleksandra Leszczynska, Wanjun Yang, Chao-Wei Hwang, Gary Gerstenblith, Andreas S. Barth, Peter V. Johnston

**Affiliations:** 1 Department of Medicine, Division of Cardiology, Johns Hopkins University School of Medicine, Baltimore, MD USA; 2 Department of Medicine, Section of Cardiovascular Medicine, Yale University School of Medicine, New Haven, CT USA; 3 Department of Pediatrics, University of California San Diego School of Medicine, San Diego, CA USA

**Keywords:** Cell signalling, Ageing, Autophagy, Pluripotent stem cells

## Abstract

Age-related differences in stem-cell potency contribute to variable outcomes in clinical stem cell trials. To help understand the effect of age on stem cell potency, bone marrow-derived mesenchymal stem cells (MSCs) were isolated from young (6 weeks) and old (18–24 months) mice. HUVEC tubule formation (TF) induced by the old and young MSCs and ELISA of conditioned media were compared to one another, and to old MSCs after 7 d in indirect co-culture with young MSCs. Old MSCs induced less TF than did young (1.56 ± 0.11 vs 2.38 ± 0.17, *p* = 0.0003) and released lower amounts of VEGF (*p* = 0.009) and IGF1 (*p* = 0.037). After 7 d in co-culture with young MSCs, TF by the old MSCs significantly improved (to 2.09 ± 0.18 from 1.56 ± 0.11; *p* = 0.013), and was no longer different compared to TF from young MSCs (2.09 ± 0.18 vs 2.38 ± 0.17; *p* = 0.27). RNA seq of old MSCs, young MSCs, and old MSCs following co-culture with young MSCs revealed that the age-related differences were broadly modified by co-culture, with the most significant changes associated with lysosomal pathways. These results indicate that the age-associated decreased paracrine-mediated effects of old MSCs are improved following indirect co-culture with young MSC. The observed effect is associated with broad transcriptional modification, suggesting potential targets to both assess and improve the therapeutic potency of stem cells from older patients.

## Introduction

The overriding goals of stem cell therapy are to improve the repair of damaged tissues and limit the loss of function following an injury. Older patients, with increased likelihood of disease, stand to benefit the most from stem cell and other regenerative therapies. Yet, advanced age and age-associated comorbid medical conditions such as diabetes and heart failure have been shown to negatively affect the therapeutic potency of a patient’s stem cells^1–9^, which may explain, in part, the variable effects of autologous cells in clinical trials to date^10–12^. At the same time, there is a growing consensus that stem cells exert beneficial effects to a large extent by release of growth factors, cytokines, exosomes, and other micro-vesicles, which are collectively referred to as “paracrine factors” (PFs)^13–15^. Age-related differences in PF composition and release may explain variations in stem cell therapeutic potency among patients whose ages differ^16–18^. However, there is also evidence that stem cells obtained from older animals can revert to a younger phenotype (i.e. be “rejuvenated”) in some cases following exposure to media conditioned by younger cells^19–21^, offering hope that stem cell tissue repair can be enhanced in those with sub-optimal cells. Less is known regarding how such “rejuvenated” old cells are modified on a transcriptional level. Understanding the effect of exposure to media conditioned by young cells on PF release by rejuvenated old cells, as well as the underlying mechanism, could lead to methods to improve cell and tissue repair in older patients.

Mesenchymal stem cells (MSCs) are frequently tested in clinical trials of cell therapy for the heart and other organs and release a spectrum of PFs^22–25^. To assess the effect of age on MSC therapeutic potency, bone marrow-derived MSCs were isolated from young (4–6 weeks) and old (18–24 months) mice and their abilities to stimulate paracrine-mediated angiogenesis were compared. We further sought to determine whether exposure of MSCs obtained from older animals to media conditioned by MSCs from young animals improves experimental angiogenic performance and PF release by the “rejuvenated” old MSCs. Further, to examine underlying differences in gene expression between young and old MSCs, and the effects of exposure of old MSCs to media conditioned by young MSCs on gene expression, we performed RNA sequencing and quantitative RT-PCR.

## Results

### MSC characterization and differentiation

**Fig. 1:**
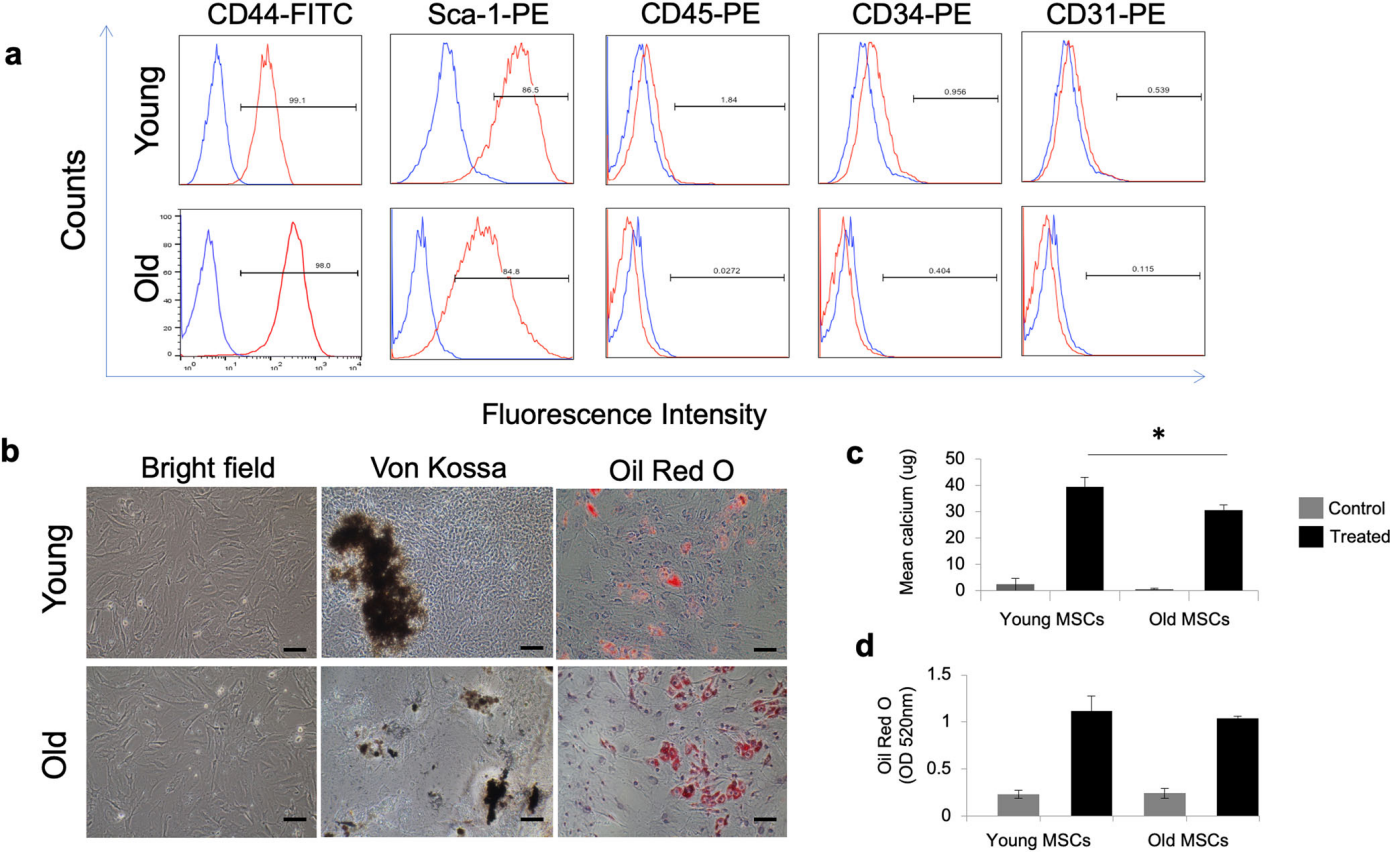
MSC characterization and in vitro differentiation capacity. **a** Flow cytometry results show that MSCs from both young and aged donor mice were uniformly positive for MSCs markers CD44 and Sca-1 and negative for hematopoietic stem cells markers CD34 and CD45 and endothelial marker CD31 (*n* = 6). **b** MSCs from young and aged donor mice display fibroblast-like morphology on bright field microscopy (left panels; scale bar = 20 μm). Following exposure to differentiation media MSCs from both young and aged mice showed osteogenic (middle panels) and adipogenic (right panels) differentiation as detected by Von Kossa and oil red O staining, respectively. **c** Quantitative assessment of calcium deposition showed significantly less calcium deposition in aged MSCs compared to young following exposure to differentiation media (*p* ≤ 0.05; *t* test; *n* = 3 each group). **d** There was no significant difference in lipid content between groups (*t* test; *n* = 3 each). MSCs cultured in control medium served as negative control for both conditions. Error bars show s.d.

Surface marker expression confirmed MSC phenotype as evidenced by expression of CD44 and SCA-1 with the absence of CD34, CD45, and CD31 (Fig. 1a). There was no significant difference in surface marker expression between old and young MSCs. Old and young MSCs were induced to undergo osteogenic and adipogenic differentiation upon exposure to respective differentiation medium (Fig. 1b). Van Kossa staining revealed the presence of mineralized matrix in all samples treated with osteogenic supplements. The staining was more prominent in young MSCs compared to old MSCs, and calcium levels were substantially greater in young, compared to old, MSCs (Fig. 1c). The formation of lipid droplets in young and aged MSCs after exposure to adipogenic media was observed using Oil Red O staining. There was no significant difference in adipogenic differentiation between the old and young MSCs (Fig. 1d).

### Young MSCs show superior paracrine-mediated angiogenesis compared to old MSCs

**Fig. 2:**
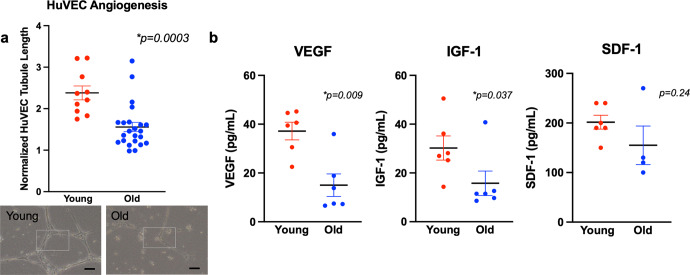
HUVEC tubule formation and relevant PF release: young v old. **a** Young MSCs housed in bioreactor tubes (*n* = 10) stimulated significantly greater TF by HUVECs over 18 h than did the same number of old MSCs (*n* = 23; *p* < 0.001; permutation test; scale bar = 20 μm). **b** Conditioned media from young MSCs produced significantly greater amounts of VEGF (*p* = 0.009; Mann–Whitney; *n* = 6 for each group) and IGF-1 (*p* = 0.047; Mann–Whitney; *n* = 6 for each group) than did conditioned media from old MSCs; there was no significant difference in SDF-1 production (Mann–Whitney; *n* = 6 for young, *n* = 4 for old). Error bars show s.e.m.

Bioreactor tubes containing 10^5^ young MSCs (*n* = 10) induced significantly greater paracrine-mediated angiogenesis as assessed by normalized tubule formation in the HUVEC assay than did BT containing the same number of old MSCs (*n* = 23): 2.38 ± 0.17 vs 1.56 ± 0.11, *p* = 0.0003 (Permutation test; Fig. 2a). Young MSCs also released significantly greater amounts of VEGF and IGF1 compared to those released by old MSCs (Fig. 2b). For VEGF: 37.2 ± 3.6 vs 15.0 ± 6.1 pg/ml (*p* = 0.009; *n* = 6 in each group; Mann–Whitney) and for IGF1: 30.2 ± 5.0 vs 15.8 ± 5.0 pg/mL (*p* = 0.037; *n* = 6 in each group; Mann–Whitney). There was no significant age difference in SDF1 release, 201.7 ± 14.*0* vs 155.0 ± 31.7 pg/ml for young and old, respectively (*p* = 0.24 *n* = 6 for young MSCs, *n* = 4 for old; Mann–Whitney).

### Rejuvenation of old MSCs by exposure to PFs released by young MSCs

**Fig. 3:**
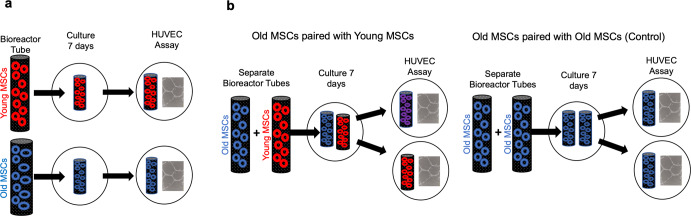
Workflow for human umbilical vascular endothelial cell (HUVEC) angiogenesis experiments. **a** The same number of MSCs obtained from old or young C57BL mice were placed in separate bioreactor tubes and grown at standard culture conditions. After 7 d the bioreactor tubes were placed separately in plates containing HUVECs in media without any supplement. Tubule formation (TF) was assessed after 18 h. **b** For the rejuvenation experiments, two separate bioreactor tubes were placed in the same well for 7 d. Pairings included old MSCs grown with separate old MSCs (control) and old MSCs grown separately with young MSCs (rejuvenation). After 7 d the bioreactor tubes were removed and used separately to stimulate HUVEC angiogenesis with TF assessed after 18 h.

**Fig. 4:**
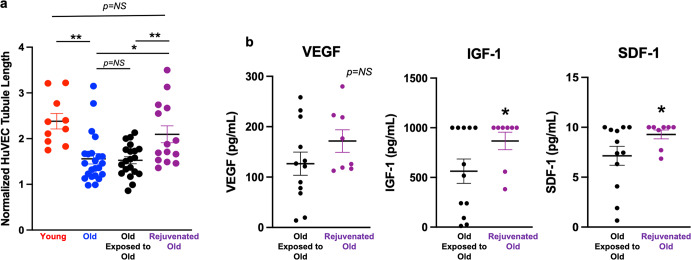
Rejuvenating effect of exposure of old MSCs to young MSCs on HUVEC tubule formation and PF release. **a** Co-culture of old MSCs with young MSCs housed in a separate bioreactor tube for 7 d (“Old × Young”; *n* = 14 in each group) “rejuvenated” the old MSCs. TF stimulated by the rejuvenated old MSCs was significantly greater than that of unexposed old MSCs and that of old MSCs grown in co-culture for 7 d with other old MSCs (“Old × Old”; *n* = 12) and was not significantly different from that of the young MSCs. **b** The rejuvenated old MSCs also produced significantly greater amounts of IGF-1 (*p* < 0.05; Mann–Whitney) and SDF-1 (*p* < 0.05; Mann–Whitney) than did old MSCs paired with other old MSCs; there was no significant difference in the release of VEGF (*p* = NS; Mann–Whitney). For figures: *p < 0.05, **p ≤ 0.01. Error bars show s.e.m.

To determine whether exposure of old MSCs to media conditioned by young MSCs improves PF-mediated angiogenesis by the older cells, bioreactors containing 10^5^ old MSCs were co-incubated with separate bioreactors containing 10^5^ young MSCs for 7 days (Fig. 3b). Control experiments were performed using 2 separate bioreactors, both containing 10^5^ old MSCs. Exposure of old MSCs to the PFs from the young MSCs significantly improved their ability to induce paracrine-mediated tubule formation in the HUVEC assay, with normalized tubule formation of 2.09 ± 0.18 (*n* = 14) by the “rejuvenated” old MSCs vs 1.56 ± 0.11 stimulated by old MSCs grown alone (*n* = 23; *p* = 0.013; Permutation test; Fig. 4a). In addition, tubule formation by the “rejuvenated” old MSCs and young MSCs grown alone were not significantly different: 2.09 ± 0.18 (*n* = 14) vs 2.38 ± 0.17 respectively (*n* = 10 in each group; *p* = 0.27; Permutation test). Tubule formation by bioreactors containing old MSCs exposed to bioreactors containing other old MSCs (*n* = 20) was significantly less than that by the “rejuvenated” old MSCs: 1.53 ± 0.08 vs 2.09 ± 0.18, respectively (*p* = 0.003; Permutation test) and not significantly different from tubule formation stimulated by bioreactors containing old MSCs grown alone: 1.53 ± 0.08 vs 1.56 ± 0.11, respectively (*p* = 0.83; permutation test).

### Improved PF release by rejuvenated old MSCs

There was a non-significant trend for higher VEGF production by the “rejuvenated” old MSCs (*n* = 8) compared to VEGF production by old MSCs grown with other old MSCs (*n* = 12 in each group): 171.5 ± 22.4 vs 126.8 ± 23.0 pg/mL (*p* = 0.17; Mann–Whitney; Fig. 4b). However, there were significant increases in their production of IGF1: 867.4 ± 88.4 vs 563.5 ± 122.6 pg/mL (*p* = 0.047; Mann–Whitney) and SDF1: 9.3 ± 0.45 vs 7.1 ± 0.95 ng/mL (*p* = 0.038; Mann–Whitney). These results indicate that IGF1 and SDF1 may play an important role in mediating the “rejuvenation” angiogenesis effect of exposure of old MSCs to media containing PFs elaborated by young MSCs.

### RNA sequencing reveals marked differences among young, old, and rejuvenated old MSCs

**Fig. 5:**
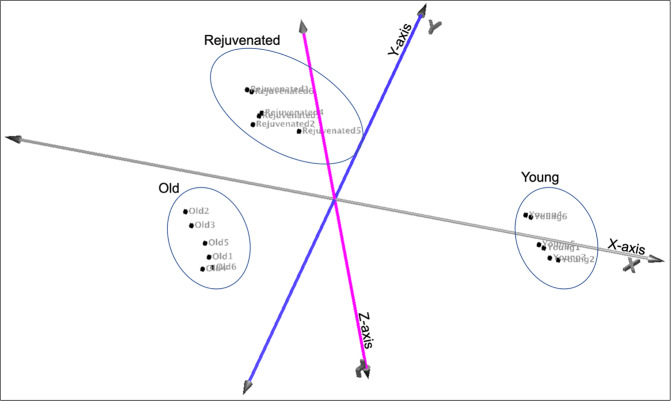
Principal component analysis (PCA) of > 20,000 analyzed genes. PCA of young, old, and rejuvenated old MSCs (6 replicates for each condition) reveals three distinct gene expression patterns.

**Fig. 6:**
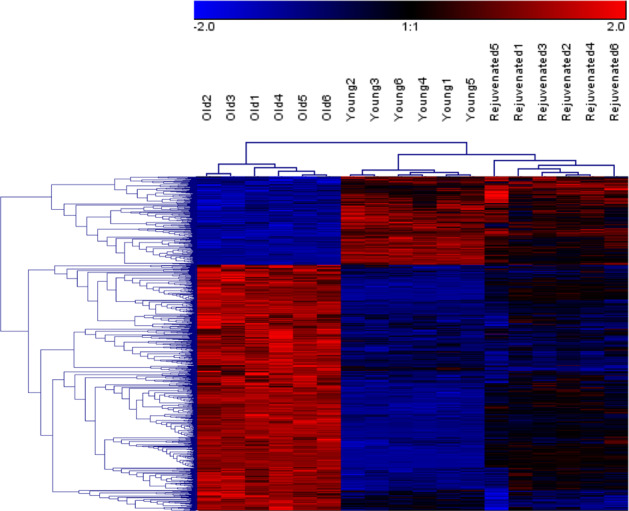
Differentially expressed genes (DEGs) with more “youthful” expression in rejuvenated old MSCs. Of the 2831 observed DEGs between the old and the rejuvenated old MSCs, 831 of those in the rejuvenated MSCs had more “youthful” expression, i.e. expression of DEGs that was more like that of the young MSCs, than that of the old MSCs. Gene expression levels are color-coded with blue and red representing low and high expression levels, respectively.

To both assess the transcriptional differences between young and old MSCs, and the changes in old MSCs mediated by exposure to media conditioned by PFs released by young MSCs, RNA sequencing was performed. Principal component analysis (PCA) of the >20,000 transcripts assessed revealed three distinct expression groups correlating with young, old, and rejuvenated old MSCs (Fig. 5). There were 2831 differentially expressed gene transcripts (DEGs) when those from the old MSCs were compared to those from the rejuvenated old MSCs. Of these, 831 were differentially expressed in rejuvenated old MSCs in a pattern that was more similar to that of the young MSCs, i.e. represented a more “youthful” expression profile (Fig. 6).

### Functional annotation

**Fig. 7:**
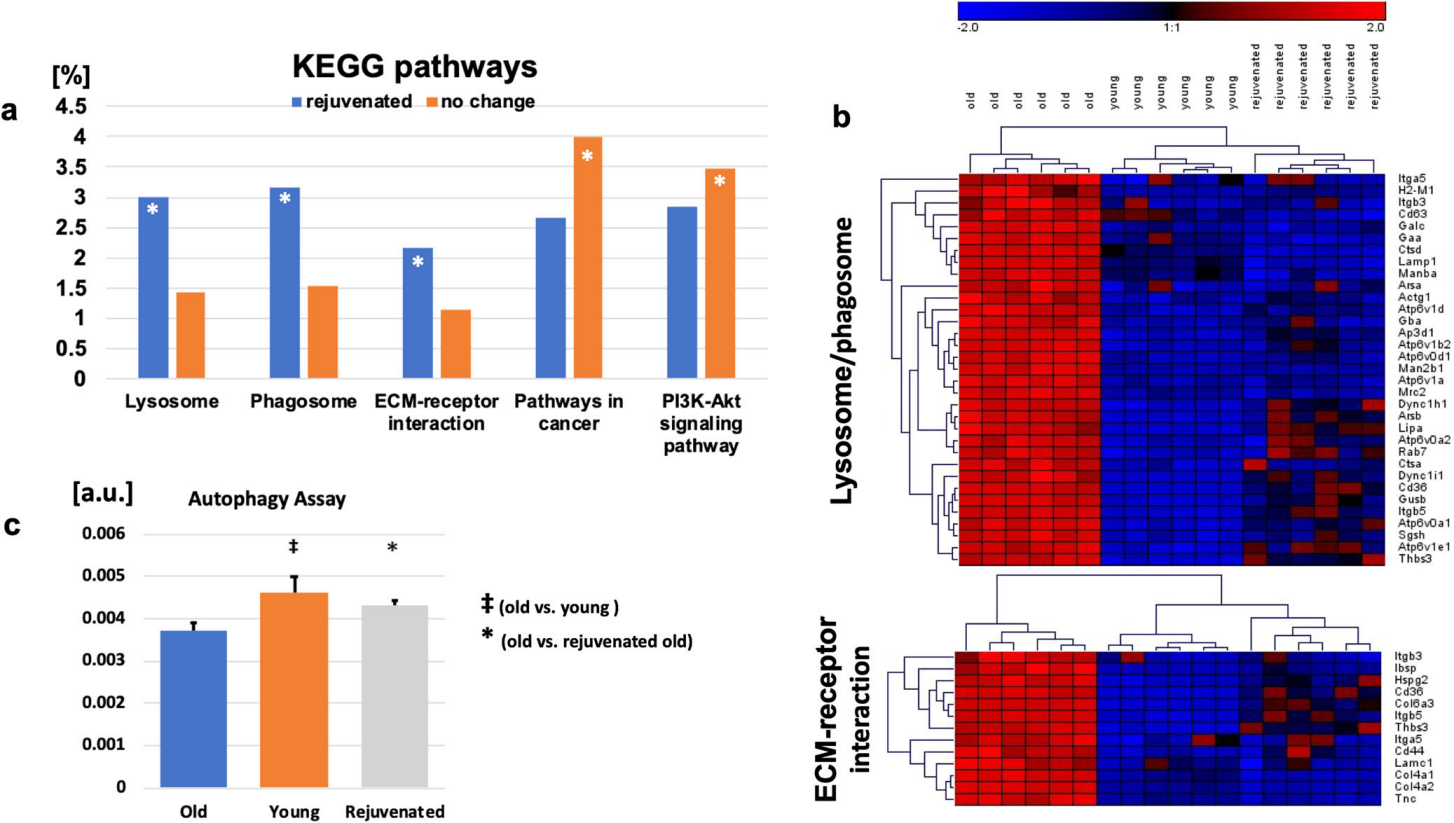
KEGG pathway analysis shows more youthful expression of lysosomal pathways in rejuvenated old MSCs. **a** Over-represented functional categories based on KEGG pathways of the 831 transcripts associated with a more youthful expression profile (* indicates false discovery rate FDR < 5%). **b** Hierarchical clustering of genes of the lysosome/phagosome (upper panel) and ECM–receptor interaction (bottom panel). Gene expression levels are color-coded with blue and red representing low and high expression levels, respectively. Expression levels of the rejuvenated old cells cluster with those of the young MSCs rather than those with the old cells. **c** Results from a functional autophagy assay comparing old, young, and rejuvenated old MSCs showed significant differences between old and young MSCs (^**‡**^*p* < 0.05; *t* test) and old vs rejuvenated old MSCs (*p < 0.05; *t* test). [a.u.] indicates arbitrary units. Error bars show s.e.m.

**Fig. 8:**
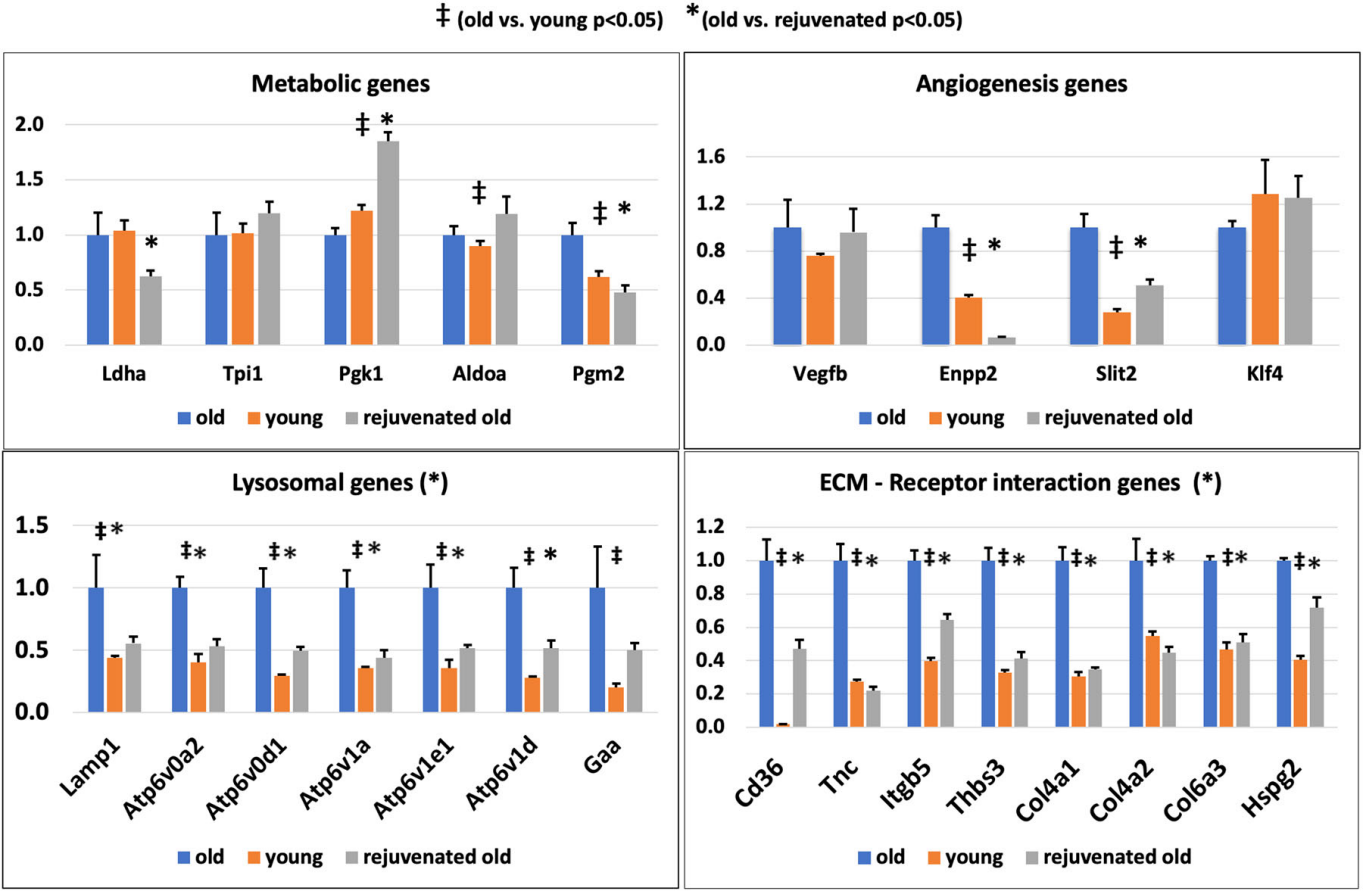
RT-PCR of selected transcripts confirms more youthful expression of rejuvenated MSCs. rtPCR of selected transcripts identified as having more youthful expression in RNA sequencing analysis showed broad agreement, with 20/24 factors showing more youthful expression in rejuvenated MSCs compared to old MSCs when normalized to housekeeping gene 18S. Selected transcripts include those associated with metabolism, ECM–Receptor interaction, autophagy / lysosome, and angiogenesis. ^‡^*p* < 0.05 for old vs. young (*t* test). *p < 0.05 for old vs. rejuvenated (*t* test). Error bars show s.e.m.

KEGG pathway analysis of the 831 DEGs with the more youthful expression profile showed that rejuvenation was associated with downregulation of lysosomal pathways and significant changes in extracellular matrix–receptor interaction (FDR < 5%; Fig. 7a, b). Several lysosomal marker genes showed significant upregulation with aging and downregulation with rejuvenation (e.g. Lamp1, Gaa, and Atp6v0a2; Fig. 7b) and expression of these genes was validated by SybrGreen RT-PCR (Fig. 8). Transcripts that showed no significant change in rejuvenation were more likely to be involved in “Pathways in cancer” and signaling pathways, e.g. the PI3K-Akt-pathway (FDR < 5%; Fig. 7). To validate the findings of the KEGG pathway analysis, functional assessment of autophagy was performed using old, young, and rejuvenated old MSCs (Fig. 7c) showing significant differences between old and young (*p* < 0.05; *t* test) and old and rejuvenated old MSCs (*p* < 0.05; *t* test) and no significant difference between young and rejuvenated old MSCs.

### rtPCR confirms more “youthful” expression

To confirm the more youthful expression observed in RNA sequencing analysis, we performed SybrGreen RT-PCR of 24 transcripts, selected for their association with lysosomal pathways, angiogenesis, ECM, and metabolism. When normalized to 18S, 20/24 differentially expressed genes showed expression in rejuvenated old MSCs more similar to that of young MSCs than that of old MSCs (Fig. 8).

## Discussion

Older patients stand to benefit greatly from regenerative therapies for the heart and other organs, although achieving the desired therapeutic effects with autologous cells may be limited by variable cell potency. These limitations may also apply to cells from those with chronic medical conditions such as diabetes and heart failure, which are also known to negatively affect stem cell potency^10–12,26^. Our work described here suggests that donor age negatively affects MSC paracrine-mediated angiogenesis as measured by tubule formation in the HUVEC assay: i.e. angiogenesis stimulated by PFs from MSCs obtained from old mice was significantly less than that stimulated by PFs from MSCs obtained from young mice. In addition to the differences observed in tubule formation, significant age-related differences in the production of key pro-angiogenic PFs (VEGF, IGF-1) were observed. These findings are consistent with prior reports that the therapeutic potency of stem cells from old animals and humans is inferior to that of those young animals and humans^2,10,12,26^. For example, in a rat model of limb ischemia, delivery of stem cells isolated from young, as compared to those from old animals resulted in higher levels of VEGF in the recipient, mobilized a greater number of endothelial progenitor cells, produced more robust angiogenesis, and increased limb perfusion^26^. Furthermore, results of studies in immunosuppressed rats post-MI demonstrated that intramyocardial injection of human MSCs from old donors is less effective than those from young donors as assessed by left ventricular ejection fraction, fractional shortening, and left ventricular cavity dimensions^2^. Similarly, bone marrow-derived hematopoietic stem/progenitor cells (HSCPCs) derived from aged mice show impaired vascular repair potential compared to those isolated from young mice, a difference that correlates with lower expression of ACE2 in the aged HSCPCs^27^.

In addition, the RNA sequencing analysis performed here reveals that underlying the significant age difference in paracrine-mediated angiogenesis are marked differences in mRNA expression, suggesting age-related dysregulation of lysosomal pathways and extracellular matrix formation. These results are important in that they could explain in part the impaired healing response in aged patients following injury, but also suggest that a uniform therapeutic effect cannot be expected when autologous stem cells are used in individuals whose ages differ. One approach to address these gaps in PF release and underlying transcriptional differences would be to use only allogeneic cells obtained from healthy young donors to treat older patients or those patients whose cells are shown to have reduced therapeutic capacity. While cells obtained from young healthy donors promise improved therapeutic potency, there remain questions regarding the risks of immune reaction and disease transmission, particularly if repeated doses are required to achieve a sustained desired effect. Our results suggest an alternative approach. Although paracrine-mediated angiogenesis by MSCs from old mice was inferior to that of MSCs obtained from young mice, this difference was abrogated by indirect co-culture of old MSCs with young MSCs. By experimental design, in which old and young MSCs were separated by semi-permeable barriers, the observed rejuvenation effect was mediated by the soluble PFs released by young MSCs.

Consistent with the observed improvement in experimental paracrine-mediated angiogenesis, the “rejuvenated” old MSCs, released significantly greater amounts of pro-angiogenic PFs as compared to controls (i.e. old MSCs exposed to other old MSCs). The increase in release of these PFs was only one manifestation of the effect elicited by exposure of old MSCs to young, however, as the numerous differences found on RNA sequencing analysis between old and rejuvenated old MSCs suggest broad transcriptional changes, the most significant of which were not directly related to angiogenesis. Functional annotation of the 831 transcripts exhibiting a rejuvenated expression profile (i.e. expression of transcripts in old MSCs that became more similar to the expression in young MSCs) revealed strong associations with genes associated with lysosomal and extracellular matrix pathways. Typically, aging is associated with dysfunction of autophagy and lysosomal pathways^28^, which are known to be significantly downregulated in MSCs isolated from old compared to young animals^29^, and our functional autophagy assay was in line with these findings. Importantly, in rejuvenated cells, autophagy was partially restored to levels seen in young cells. Interestingly, we noticed a discordance between functional autophagy assay and mRNA levels, but the prominent upregulation of transcripts associated with lysosomal autophagy in aged cells could represent a compensatory mechanism to improve lysosomal degradation. This unexpected finding suggests there is much we do not understand about the rejuvenating effect of our model and may explain why not all studies of parabiosis and rejuvenation show a beneficial effect^21^. Given the mixed transcriptional effect on lysosomal/autophagy genes in aging and following rejuvenation, further studies of lysosomal function are necessary.

Besides reversal of age-associated changes in lysosomal pathways/autophagy, our analysis also identified extracellular matrix–receptor interaction, as a critical pathway involved in the observed rejuvenation of old MSCs, including dysregulation of collagen 4 (Col4a1, Col4a2) and integrins (Itga5, Itgb3, Itg5). Prior studies have shown a close link between ECM remodeling and autophagosomes. For example, mice deficient in lysosome-associated membrane protein-2 (Lamp2), a glycoprotein that plays a critical role in lysosomal biogenesis and maturation of autophagosomes/phagosomes, showed a reduction in ECM proteins collagen 4, laminin, and fibronectin^30^, consistent with the parallel regulation of ECM and lysosomal pathways observed in our study. Cell-matrix interactions are mediated primarily by integrins, which are membrane-spanning receptors capable of cell or matrix component recognition and are involved in intracellular signal transduction. Aging is associated with quantitative and qualitative changes in matrix macromolecules and the integrins that bind them^31^. Importantly, there is a close interaction between beta3 and beta1 integrins and growth factors responsible for the control of angiogenesis^32^. Further, age-related impairment of cross-activation of β3 integrin and paracrine factors in endothelial progenitor cells was previously shown to decrease angiogenesis^33^, similar to the findings we report here.

In summary, our findings thus show there are significant age-related differences in paracrine-mediated angiogenesis that are associated with significant differences in lysosomal and extracellular matrix regulatory pathway expression. The observed differences may explain in part the variable effects reported from clinical studies using autologous MSCs and other stem cells in older patients, but also suggest potential strategies to overcome these limitations. First, autologous cells obtained from older patients could be grown ex vivo in co-culture with young, therapeutically-optimal cells for a period of time (e.g. 7 days) prior to returning these cells to the patient. Importantly, the “rejuvenating” effect observed following exposure of old MSCs to young MSCs was indirect and mediated by release of soluble paracrine factors as opposed to direct cell-cell interaction, as old and young MSCs were always separated by a semi-permeable membrane. The results suggest that identification of the combination of particular PFs responsible for the rejuvenating effect using metabolomic comparison of media conditioned by old vs young MSCs could allow for the development of a “rejuvenation” media designed to improve the therapeutic efficacy of older stem cells without requiring co-culture with other cells. Further work is necessary to examine this effect in vivo, although prior studies support the applicability of these strategies^11,20,34–36^.

A second strategy would be to harness the observed transcriptional modifications that accompanied improved angiogenic performance in rejuvenated old MSCs to identify modifiable genes that could be targeted to coax cells from older patients toward a younger expression phenotype^37^. In particular, the 831 DEGs that exhibited a more “youthful” expression profile in rejuvenated old MSCs include both upregulated and downregulated transcripts linked to several transcription factors that could be therapeutic targets. Markers of a more “youthful” expression profile, particularly more youthful expression of lysosomal pathway/autophagy genes, might also be used to identify older patients whose cells may not require “rejuvenation”. Chronologic age alone does not determine relative stem cell potency, and there may be patients who, despite being of advanced age, have stem cells with therapeutic quality sufficient to generate the desired clinical effect^10^. For example, gene expression profiling along with assessment of ex vivo PF release (e.g. VEGF, IGF, SDF1) could be used to assess the expected therapeutic effect of autologous stem cells and to design criteria to determine whether cellular rejuvenation would be of benefit. While such assessment has not been used prospectively to improve regenerative therapies, results from the PERFECT trial of CD133 + hematopoietic stem cells in patients undergoing coronary artery bypass surgery did show that pre-operative gene expression profiling could predict the likelihood of a therapeutic effect following intramyocardial injection of autologous hematopoetic stem cells at the time of open-heart surgery^38,39^. In addition, the CardiAMP Heart Failure Trial of autologous bone marrow mononuclear cells in patients with symptomatic heart failure due to ischemic cardiomyopathy includes a cell potency assay that is used to screen study participants and exclude those whose cells have markers suggestive of reduced therapeutic efficacy based on prior clinical trials^40,41^. The results of this multi-center randomized controlled study will be important to help determine whether screening therapeutic potency of autologous cells is of clinical value^12^.

In summary, the experimental results reported here suggest that paracrine-mediated angiogenesis by old MSCs is inferior to that of young MSCs. The observed age-related difference is associated with significant differences in the release of pro-angiogenic PFs and marked differences in gene expression, particularly those related to lysosomal pathways. These differences are not fixed, however, and exposure of old MSCs to soluble PFs released by young MSCs in culture significantly improved paracrine-mediated angiogenesis by “rejuvenated” old MSCs with significant differences in PF-release and broad underlying transcriptional modification. These results suggest potential strategies that could be used to screen and/or “rejuvenate” stem cells from older patients, to allow for a more predictable effect when autologous cells are used clinically. It will be important to understand differences in stem cell therapeutic potency mediated by age and chronic medical conditions (diabetes, hypertension, smoking, etc.) in order to optimize stem cell therapy for the benefit of older patients and others whose regenerative capacities may be limited, as these patient populations are those who arguably stand to benefit most from regenerative therapeutic strategies.

## Methods

### Mesenchymal stem cell isolation and expansion

Bone marrow-derived MSCs were isolated from “young” (6 weeks) and “old” (18–24 months) C57 black male mice using established techniques^42,43^ under a protocol approved by the Johns Hopkins University Animal Care and Use Committee. Briefly, immediately following euthanasia, whole bone marrow was flushed out from the bilateral tibias and femurs. After washing by centrifugation at 400 *g* for 10 min, cells were plated at 5 × 10^6^ viable cells per ml. The culture was kept in humidified 5% CO_2_ incubator at 37 °C for 72 h, when non-adherent cells were removed by changing the media.

### MSC characterization

All MSC preparations were evaluated using flow cytometry with PE or FITC-conjugated antibodies against murine Sca-1 (1:200; BioLegend 122507), CD31 (1:200; Fisher Scientific BDB554473), CD34 (1:100; eBioscience 14-0341-82), CD44 (1:100; BioLegend 103007), CD45 (1:100; BioLegend 103105), and IgG (1:100; BioLegend 400607) performed on BD LSRII (Becton Dickinson) using DIVA software. At least 10000 events were collected. FlowJo software was used to analyze and create the histograms.

### Ostenogenic and apidogenic differentiation

Assessment for osteogenic and adipogenic differentiation was performed using established techniques^43^. Briefly, to induce osteogenic differentiation, old and young MSCs were seeded into 6-well plates at 1.3 × 10^4^ cells/well. After 24 h the media was replaced with osteogenic differentiation medium containing Iscove’s medium supplemented with 100 nM dexamethasone, 10 mM beta-glycerophosphate, 50 μM ascorbic acid, and 1% antibiotic/antimycotic. Cells were maintained in induction media with media changes every 2 days. After 14 days cells fixed in 10% formalin for 15 min and calcium deposition was assessed using von Kossa staining. Calcium deposition was then quantified using a colorimetric calcium assay (Calcium CPC Liquicolour Kit StanBio, Boerne, TX) according to the manufacturer’s instructions. To induce adipogenic differentiation, old and young MSCs were seeded in 6-well plates at 2 × 10^5^ cells/well. When confluent the media was replaced with adipogenic induction medium containing DMEM-HG, 10% FBS, 5% rabbit serum, 1uM dexamethasone, 10 μg/mL insulin, 200 μM indomethacin, 500 μM isobutylmethylxanthine (IBMX), and antibiotic/antimycotic for 3 days followed by exposure to followed by exposure to adipogenic maintenance medium (DMEM-HG, 10% FBS, insulin 10 µg/ml and P/S) for 3 days. After 3 cycles of induction and maintenance exposure cells were rinsed with PBS and fixed in 10% formalin for 10 min. The cells were then stained with Oil Red O to assess for lipid droplets. After imaging Oil Red O extraction was performed using 100% isopropanol. Extract samples were transferred to a 96-well plate and absorbance readings were taken at 490 nm to quantify extracted Oil Red O.

### MSC culture and expansion

Confirmed MSCs were expanded in culture in media prepared by combining 490 ml Medium 200 PRF (Gibco Invitrogen, Carlsbad, CA), a standard basal medium intended for culture of large vessel human endothelial cells, with 10 ml Low Serum Growth Supplement (LSGS; Gibco Invitrogen). The final preparation contained 2% fetal bovine serum (FBS), 3 ng/ml basic fibroblast growth factor (bFGF), 10 ng/ml human epidermal growth factor, 10 μg/ml heparin, and 1 μg/ml hydrocortisone. Cells were incubated under standard conditions (5% CO_2_ and 37 °C). Expanded MSCs at low passage numbers (P2-P5) were used for the experiments. In the event frozen cells were used, they were thawed and grown for one passage prior to use in the experiments.

### Bioreactor tube preparation

To prevent cell-cell interaction and assess only paracrine-mediated effects (i.e. those resulting from release of soluble factors), angiogenesis experiments were performed using bioreactor tubes (BT) constructed with CellMax semi-permeable polysulfone membrane tubing (Spectrum Labs, Rancho Dominguez, CA). These allowed the free diffusion of soluble proteins and other molecules released by the cells up to a 500 kDa molecular weight cut-off, but not of the cells themselves. To load BTs, MSCs were trypsinized and suspended in Medium 200 PRF without LSGS supplementation (i.e. media devoid of stimulatory growth factors). MSCs were counted using a Scepter automated cell counter (Millipore, Billerica, MA), which had been previously standardized for accuracy. The desired number of MSCs was spun down and resuspended to a total volume of 100 ul that was injected into the BTs using a 0.5 mL syringe. To compare paracrine-mediated angiogenesis by old and young MSCs, BTs were loaded with either 10^5^ old or 10^5^ young MSCs. Once cell injection was complete, the tubes were heat-sealed at both ends and the MSC-loaded tubes, fully submerged in media, were grown at standard culture conditions (37 °C, 5% CO_2_) for 7 days (Fig. 3a).

### ELISA assay for paracrine factor release

ELISA assays were performed to measure paracrine factor (PF) production by the MSCs contained within the BTs grown in culture. Tubes loaded with 2 × 10^5^ MSCs were submerged in 5 mL of alpha-MEM basal medium (Stemcell Technologies, Tukwila, WA) supplemented with 20% FBS (Gibco Invitrogen, Carlsbad, CA) in a 6-well plate. At day 7, conditioned media was collected from each well, spun down for 1 min to pellet any debris, and then flash frozen at −80 °C. Conditioned media samples were assessed for the concentrations of vascular endothelial growth factor (VEGF), stromal derived factor-1 (SDF1) and insulin-like growth factor-1 (IGF1) by ELISA (Quantikine, R&D Systems, Minneapolis, MN) according to the manufacturer’s instructions.

### HUVEC assay

BTs were removed at day 7 and placed in separate wells of a 6-well plate containing human umbilical vein endothelial cells (HUVECs)^44^. Briefly, 10^5^ HUVECs (Gibco Invitrogen, Carlsbad, CA) suspended in Medium 200PRF were plated per well in Geltrex (Gibco Invitrogen) coated 6-well plates. Negative control wells received a bioreactor loaded with un-supplemented Medium 200PRF only (i.e. no cells). Positive control wells were plated with 10^5^ HUVECs suspended in 1 mL of Medium 200PRF supplemented with LSGS, which is known to induce HUVEC tubule formation. After 18 h at standard culture conditions (37 °C, 5% CO_2_), the wells were imaged to allow quantitative analysis of the resultant HUVEC tubule network. Images were taken in the center of each well and in all four quadrants at pre-determined locations (5 pictures total), at 100x magnification. The total length of the tubule networks captured in the images of each well was measured using ImageJ software. To allow for comparisons between experiments, the total length of the tubule network in each well was normalized to the average length of the tubule network in the negative control wells, and reported as a normalized ratio.

### Conditioning of old MSCs by young MSC PFs

To assess the effect of young MSC-generated PFs on PF-mediated angiogenesis by old MSCs, BTs were prepared as described above containing either 10^5^ young or 10^5^ old MSCs. Two BTs were placed together in a 6-well plate in 5 mL MSC media and incubated for 7 days at standard culture conditions (Fig. 3b) using a BT containing old MSCs paired with either a separate BT with other old MSCs (control) or a separate BT with young MSCs. After 7 days the tubes were removed, washed with un-supplemented Medium 200 PRF, and then used separately in the HUVEC assay as described above. After the HUVEC assay was complete (18 h) the BTs were placed in separate wells of 6-well plates and grown in culture for 7 additional days with collection of conditioned media for PF release.

### RNA sequencing and RT-PCR of old MSCs and rejuvenated old MSCs

Replicates of 10^5^ old MSCs were cultured separately, or in co-culture with young MSCs, for 7 days using a 0.4 µm Transwell system in 6-well plates (Corning), which allow transfer of soluble paracrine factors released by the cells, but not of the cells themselves. Following RNA purification, library preparation, amplification, and Illumina sequencing, the open source Galaxy pipeline was used for data processing and analyses. After alignment of raw sequencing reads to the UCSC mm10 genome using HISAT2, transcript assembly, alignment quantification, count normalization, and differential expression analyses were conducted with StringTie, featureCounts, DESeq2, and Genesis. Quantitative PCR (KAPA SYBR FAST One-Step qRT-PCR, Wilmington, MA) was used to validate 24 transcripts identified by RNA sequencing. Target genes were selected based on their presence in significantly regulated pathways and quantified relative to 18S ribosomal RNA using the 2^−ΔΔCt^ method^45^.

### Autophagy assay

To validate the results of the RNA sequencing and RT-PCR results, a functional autophagy assay was performed to assess relative autophagy between old, young, and rejuvenated old MSCs. Old, young and rejuvenated cells were cultured (or co-cultured, in the case of rejuvenated cells) for 7 days in 6-well plates (10^5^ cells per well). On Day 8, cells were trypsinzed, counted and 10^4^ cells were transferred to each well of a 96-well black plate with clear bottom and incubated for 6 h. The Autophagy Assay Kit (Sigma Aldrich, St. Louis, MO) measures autophagy using a proprietary fluorescent autophagosome marker in a microplate reader (λ_ex_ = 360; λ_em_ = 520 nm). Three separate experiments were performed in triplicate each for each condition. To account for possible differences introduced by counting cells, results for each cell type were normalized based on absorbance (450 nm) of a Cell Counting Kit-8 (Dojindo Molecular Technologies, Inc. Rockville, MD).

### Statistics

Data are reported as mean ± standard error of the mean (SEM) unless otherwise indicated. Comparisons between groups for the HUVEC experiments were performed using the permutation test. For the PF ELISA data, groups were compared using the Mann–Whitney test. The autophagy assay and rt-PCR results were assessed using two-tailed *t* tests. For these experiments a *p*-value < 0.05 was deemed significant. In the RNA sequencing differential expression analysis, a false discovery rate (FDR) of <0.05 was considered significant.

## Data Availability

The data that support the findings of this study are available from the corresponding author upon reasonable request. RNA seq data are available on GEO with accession number GSE205205.
